# Retinal Layers Changes in Human Preclinical and Early Clinical Diabetic Retinopathy Support Early Retinal Neuronal and Müller Cells Alterations

**DOI:** 10.1155/2013/905058

**Published:** 2013-06-12

**Authors:** Stela Vujosevic, Edoardo Midena

**Affiliations:** ^1^Department of Ophthalmology, University of Padova, Via Giustiniani 2, 35128 Padova, Italy; ^2^Fondazione G. B. Bietti, Via Livenza 3, 00198 Roma, Italy

## Abstract

*Purpose*. To evaluate the changes in thickness of individual inner and outer macular and peripapillary retinal layers in diabetes. *Methods*. 124 subjects (124 eyes) were enrolled: 74 diabetics and 50 controls. Macular edema, proliferative diabetic retinopathy (DR), any intraocular treatment and refractive error >6 diopters were the main exclusion criteria. Full ophthalmic examination, stereoscopic fundus photography, and spectral domain-OCT were performed. After automatic retinal segmentation (layering) in 5 layers, the thickness of each layer was calculated, and values compared among groups. *Results*. Thirty patients had no DR, 44 patients had non proliferative DR. A significant increase of inner plexiform and nuclear layers was found in DR eyes versus controls (*P* < 0.001). A significant decrease (*P* < 0.01) of retinal nerve fiber layer (RNFL) and at specific sites of retinal ganglion cell layer (*P* = 0.02) was documented in the macula. In the peripapillary area there were no differences between diabetics and controls. *Conclusions*. Decreased RNFL thickness and increased INL/OPL thickness in diabetics without DR or with initial DR suggest early alterations in the inner retina. On the contrary, the outer retina seems not to be affected at early stages of DM. Automatic intraretinal layering by SD-OCT may be a useful tool to diagnose and monitor early intraretinal changes in DR.

## 1. Introduction

Diabetic retinopathy (DR) is the first cause of visual impairment and blindness in the adult working-age population [1]. For a long period of time, DR has been considered primarily a retinal microvascular disorder caused by the direct effects of hyperglycemia and by the metabolic pathways it activates [2]. Nevertheless, some recent studies have demonstrated that retinal neurodegeneration (the result of a negative balance between neurotoxic and neuroprotective factors) is present even before the development of clinically detectable microvascular damage. Retinal neurodegeneration may therefore represent an early event in the pathophysiology of DR and may anticipate the onset of microvascular changes [2–4]. The term neurodegeneration used in this paper encompasses pathologic phenomena affecting both the pure neuronal component and the glial one. The hypothesis according to which neurodegeneration precedes the vascular one is confirmed by some electrophysiological and psychophysical studies, which show that the alterations are present even before the microvascular damage becomes ophthalmoscopically or angiographically visible. Such retinal function alterations mainly consist in contrast sensitivity loss, altered color perception, and failure of retinal recovery time [5, 6]. Moreover, it has been observed that in diabetic mice the oscillatory potentials of the electroretinogram (ERG) have increased peak latencies and/or reduced amplitudes, suggesting a compromised inner retinal function secondary to neuronal transmission alterations or to the combined loss of amacrine and ganglion cells [7].

During the course of DR, apoptotic cells have been observed in all retinal layers, suggesting the involvement of different types of neurons [8]. Numerous studies have evidenced that diabetes, through the alteration of different metabolic pathways, induces functional deficits and even the loss of different types of retinal cells which cover from the inner to the outer retinal cells: ganglion cells, bipolar cells, amacrine cells, horizontal cells, and eventually photoreceptors [9]. 

Different authors reported a decrease in retinal thickness in diabetic eyes with or without clinical signs of DR compared to normal subjects [10–13]. Biallosterski et al. found a significant reduction in pericentral macular thickness in 53 diabetic patients with mild nonproliferative DR [10]. Van Dijk et al. have demonstrated by spectral domain optical coherence tomography (SD-OCT) a decrease in the inner retinal thickness in the macula in diabetics with mild DR, suggesting that this phenomenon might be firstly due to ganglion cells loss in the pericentral areas and secondly to retinal nerve fiber layer (RNFL) thinning in the peripheral macula [14, 15]. 

The main purpose of this study was to identify in vivo, by SD-OCT, the changes in thickness of selected retinal layers both in the macula and the peripapillary area in diabetic patients without DR or with early stages of DR (mild and moderate nonproliferative DR) versus normal subjects.

## 2. Material and Methods

One hundred twenty-four subjects (74 diabetic patients and 50 normal subjects) were included in this study. One eye of each subject was used for the spectral domain optical coherence tomography (SD-OCT) analysis. The exclusion criteria were as follows: proliferative DR, macular edema, any type of previous retinal treatment (macular laser photocoagulation, vitrectomy, intravitreal steroids, and/or antiangiogenic drugs), any intraocular surgery, refractive error > 6D, previous diagnosis of glaucoma, ocular hypertension, uveitis, other retinal diseases, neurodegenerative disease (e.g., Alzheimer's, Parkinson's, and dementia), and significant media opacities that precluded fundus examination or imaging.

A written consent form was obtained from all patients as well as the approval from our institutional ethics committee. The study was conducted in accordance with the tenets of the Declaration of Helsinki.

Each subject underwent a complete ophthalmic examination, with determination of best corrected visual acuity, anterior segment examination, Goldman applanation tonometry, indirect ophthalmoscopy, and 90D lens biomicroscopy. Then, SD-OCT and fundus photography were obtained. 

### 2.1. Study Procedures

#### 2.1.1. Visual Acuity

Best corrected distance visual acuity (BCVA) for each eye was measured by a trained examiner using standard Early Treatment Diabetic Retinopathy Study (ETDRS) protocol at 4-meter distance with a modified ETDRS distance chart transilluminated with a chart illuminator (Precision Vision) [16]. Visual acuity was scored as the total number of letters read correctly and converted to the logarithm of the minimum angle of resolution (logMar).

#### 2.1.2. Fundus Photography

Color stereoscopic fundus photographs (7 ETDRS fields) were taken after an adequate dilatation by a trained photographer using the same TOPCON TRC 50IA 35 degree fundus camera (TOPCON, Tokyo, Japan). Diabetic retinopathy was graded as no DR and as nonproliferative DR mild or moderate DR (NPDR) by two independent graders experienced in grading DR.

#### 2.1.3. Spectral Domain OCT

All eyes were examined with spectral domain optical coherence tomography (SD-OCT, RS-3000, NIDEK, Gamagori, Japan). This instrument has a light source of 880 nm wavelength. Each eye was examined, after pupillary dilation, both in the macula and the peripapillary area. The following scanning protocols were used: “Macula Map” in the macula and “Disc Circle” in the peripapillary area. 

**Figure 1:**
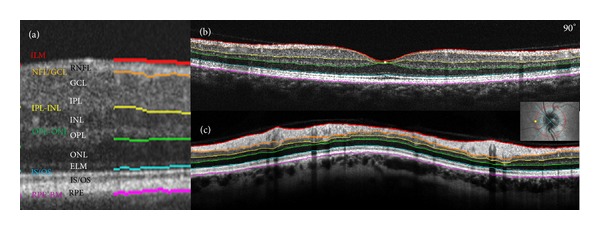
Spectral domain OCT automatic segmentation of retinal layers in the macula ((a) and (b)) and in the peripapillary area (c). In the macula the segmentation is performed on the linear scan (b) and in the peripapillary area on the circular scan around the optic disc (c). Six lines determine 5 retinal layers which from inside out are as follows: inner limiting membrane + nerve fibre layer (ILM + RNFL); ganglion cell layer + inner plexiform layer (GCL + IPL); inner nuclear layer + outer plexiform layer (INL + OPL); outer nuclear layer + external limiting membrane (ONL + ELM); and inner segment/outer segment photoreceptor layer + retinal pigment epithelium (IS/OS + RPE) (a).

The Macula Map scan pattern evaluates 6 × 6 mm area centered on the fovea with 64 horizontal B-scan lines, each consisting of 1024 A-scans per line. For each SD-OCT linear scan, an automatic algorithm has individuated 5 different retinal layers based on the different shades of gray corresponding to the reflectivity indexes of each layer, which include from inside to outside the following: inner limiting membrane + nerve fiber layer (ILM + RNFL); ganglion cell layer + inner plexiform layer (GCL + IPL); inner nuclear layer + outer plexiform layer (INL + OPL); outer nuclear layer + external limiting membrane (ONL + ELM); and inner segment/outer segment photoreceptor layer + retinal pigment epithelium (IS/OS + RPE). Retinal thickness was automatically calculated in the 9 ETDRS areas (consisting in a central circular zone with a 1-mm diameter, representing the foveal area and inner and outer rings of 3 and 6 mm diameter, resp.). The inner and the outer rings are divided into four quadrants: superior, nasal, inferior, and temporal. Mean retinal thickness and mean thickness of each of the five retinal layers in each of the nine ETDRS subfields were recorded (Figures 1(a) and 1(b)).

In the peripapillary area, a circle scan centered on the optic disc (3.46 mm diameter, “Disc Circle” option) was used. Peripapillary retinal thickness was automatically measured by the instrument in the temporal, superior, nasal, and inferior quadrants (Figure 1(c)).

If any instrument error in the automatic segmentation of retinal layers was documented, the manual correction consisted in the repositioning into proper place of the incorrectly placed points (using high magnification images), in order to redefine the retinal profile. Each grader was blinded to clinical data of all examined eyes.

#### 2.1.4. Statistics

Age, spherical equivalent, IOP, and visual acuity were compared among groups by means of analysis of variance (ANOVA); The mean values of retinal layers' thickness in each group, both in macula and peripapillary area, were confronted using the repeated measures analysis of variances (ANOVA-RM). In cases of significant results (*P* < 0.05), the ANOVA-RM was followed by the Bonferroni multiple comparisons post hoc test. All statistical analyses were performed with SAS 9.2 for Windows, SAS (Cary, NC, USA).

## 3. Results 

Of 124 enrolled subjects 74 were diabetics, (49 males and 25 females). Of 50 normal subjects, 21 were males and 29 females. Mean age of diabetics was 56.4 ± 12.7 years (range: 31–83 years); mean age of controls was 55.8 ± 13 years (range: 25–80 years). Thirty eyes were graded as no DR and 44 eyes as nonproliferative DR (NPDR). Eighteen patients (24.32%) had type 1 DM and 56 (75.68%) had type 2 DM. Mean HbA1c was 8.1% (range: 5.3%–11%). There was no significant difference in age (ANOVA, *P* = 0.98), spherical equivalent (ANOVA, *P* = 0.12), IOP (ANOVA, *P* = 0.4), and visual acuity (ANOVA, *P* = 0.5) among controls, no DR, and NPDR groups (Table 1).

**Figure 2:**
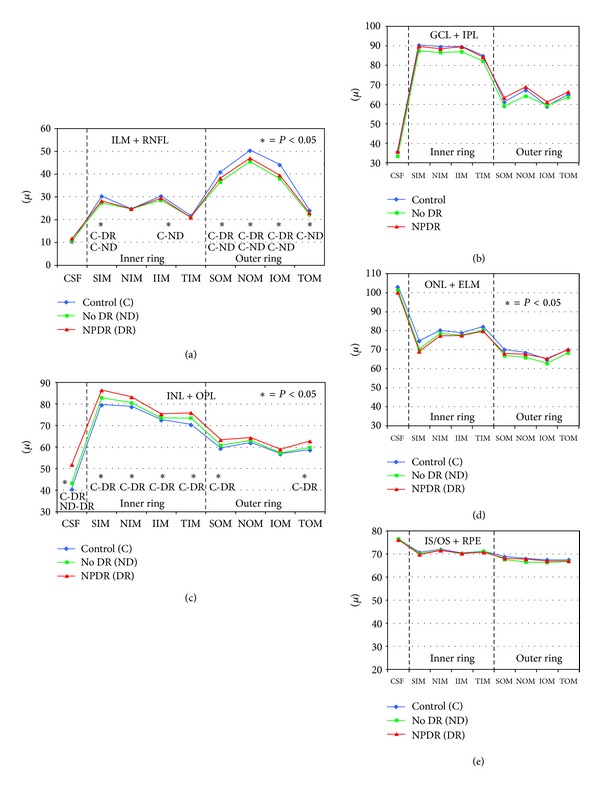
Graphs showing specific retinal layer thickness in normal subjects (control), diabetics without retinopathy (no DR), and diabetics with nonproliferative diabetic retinopathy (NPDR) determined automatically by spectral domain OCT in 9 ETDRS areas in the macula. (a) Inner limiting membrane + nerve fibre layer (ILM + RNFL); (b) ganglion cell layer + inner plexiform layer (GCL + IPL); (c) inner nuclear layer + outer plexiform layer (INL + OPL); (d) outer nuclear layer + external limiting membrane (ONL + ELM); and (e) inner segment/outer segment photoreceptor layer + retinal pigment epithelium (IS/OS + RPE). (∗) indicates statistically significant values; CSF: central subfield thickness; SIM: superior inner quadrant in the macula; NIM: nasal inner quadrant; IIM: inferior inner quadrant; TIM: temporal inner quadrant; SOM: superior outer quadrant in the macula; NOM: nasal outer quadrant; IOM: inferior outer quadrant; TOM: temporal outer quadrant.

In the macula, ILM + RNFL thickness was significantly decreased in the superior outer quadrant (SOM, *P* < 0.0001), inferior outer quadrant (IOM, *P* < 0.0001), temporal outer quadrant (TOM, *P* = 0.01), nasal outer quadrant (NOM, *P* = 0.0003), superior inner quadrant (SIM, *P* = 0.0003), and inferior inner quadrant (IIM, *P* = 0.01) in no DR group versus controls. RNFL thickness was significantly decreased in the SOM (*P* < 0.0001), NOM (*P* = 0.0003), IOM (*P* = 0.001), TOM (*P* = 0.01), and SIM (*P* = 0.003), in the NPDR group versus controls (Figure 2(a)). 

GCL/IPL thickness was not statistically significantly different between diabetics and controls, although there was a trend toward decreasing thickness in no DR group versus controls in the inner and outer rings' quadrants. GCL/IPL thickness was significantly decreased only in the NOM and SOM (*P* = 0.02, for both) in diabetics with no DR versus NPDR group (Figure 2(b)).

INL/OPL thickness was significantly increased in the central OCT subfield (CSF, *P* = 0.004), SIM (*P* = 0.003), NIM (*P* = 0.04), TIM (*P* = 0.0018), SOM (*P* = 0.002), IOM (*P* = 0.04), and TOM (*P* = 0.001) in the NPDR group versus controls (Figure 2(c)). 

There was no difference in the ONL/ELM and IS/OS − RPE thickness between diabetics with and without DR and controls (Figures 2(d) and 2(e)).

In the peripapillary area, retinal thickness was significantly decreased with increasing age (*P* = 0.0021) and in males versus females (*P* = 0.0004) in both controls and diabetics.

**Figure 3:**
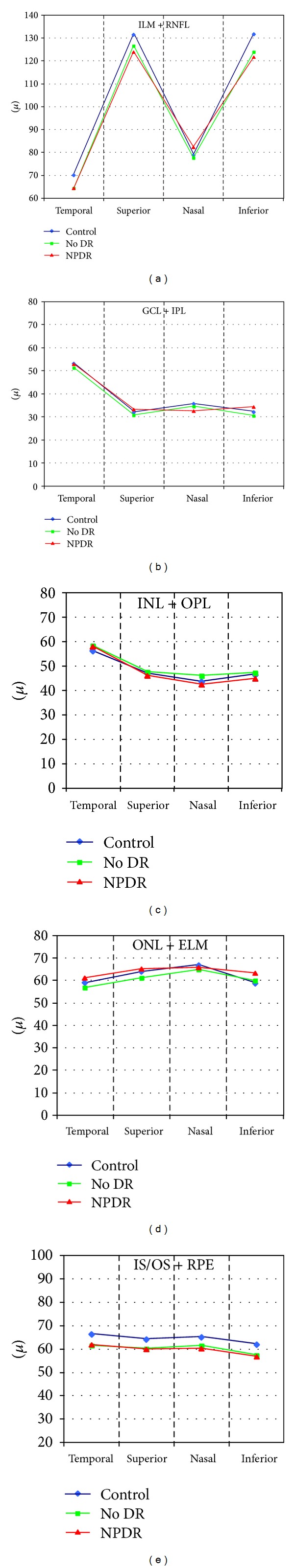
Graphs showing specific retinal layer thickness in normal subjects (control), diabetics without retinopathy (no DR), and diabetics with nonproliferative diabetic retinopathy (NPDR) determined automatically by spectral domain OCT in 4 peripapillary areas (temporal, superior, nasal, and inferior). (a) Inner limiting membrane + nerve fibre layer (ILM + RNFL); (b) ganglion cell layer + inner plexiform layer (GCL + IPL); (c) inner nuclear layer + outer plexiform layer (INL + OPL); (d) outer nuclear layer + external limiting membrane (ONL + ELM); and (e) inner segment/outer segment photoreceptor layer + retinal pigment epithelium (IS/OS + RPE). There is no significant difference in the retinal layer thickness among the controls, no DR, and NPDR groups.

 ILM + RNFL thickness was significantly different in 4 quadrants, thicker in the superior and inner quadrants, and thinner in the nasal and temporal quadrants. There was no significant difference in the ILM + RNFL, GCL/IPL, INL/OPL, ONL/ELM, and IS/OS − RPE thickness between controls and diabetics (Figure 3). 

## 4. Discussion

**Figure 4:**
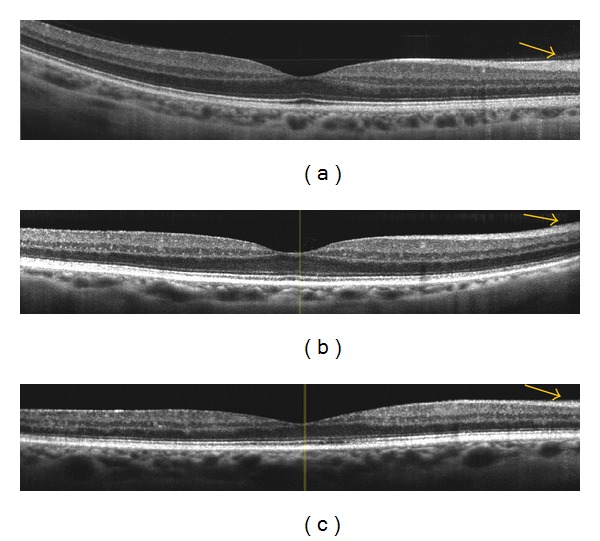
Spectral domain OCT linear scans in the macula of (a) normal subject, (b) diabetic patient without retinopathy, and (c) diabetic patient with mild nonproliferative diabetic retinopathy. The arrows indicate a progressive thinning of the retinal nerve fiber layer in diabetics (without and with retinopathy) versus normal subjects.

In this study we report a decrease in RNFL thickness in the macula of diabetic eyes even without any clinical sign of retinopathy (Figure 4). Reduced RNFL thickness may be explained by progressive ganglion cells and astrocytes loss induced by diabetes. It may depend on a direct toxicity of hyperglycemia or on Müller cells dysfunction, which are unable to maintain an adequate osmotic equilibrium between the intra- and the extracellular matrices with consequent apoptosis of neuronal cells and progressive axonal degeneration [2, 9, 17–20]. Different authors have reported the thinning of RNFL and, in some cases, of the GCL + IPL complex, suggesting that retinal neurodegeneration is an early event in diabetes mellitus, representing a preclinical stage of DR [15, 21, 22]. In fact, the decrease of RNFL thickness in the superior macular region in diabetics without DR or with minimal signs of DR has been documented in vivo [23–26]. Lonneville et al. have demonstrated that RNFL thickness decreases with poor metabolic control in diabetics with or without clinically detectable DR [27]. In this study, we did not find significant difference in RNFL thickness between diabetics without DR and with NPDR. This is probably due to the fact that all our patients were at early stages of DR and had quite good metabolic control. In this study we did not find statistically significant difference in the thickness of GCL/IPL between diabetics and controls, although there was a trend of decreasing GCL/IPL thickness in diabetics without DR versus controls in the pericentral macula. This may be due to the small differences in the specific layer thickness, thus suggesting a more numerous study population. 

The INL and the OPL showed increased thickness in diabetic patients with NPDR versus controls in this study. The INL is mainly formed by the nuclei of bipolar and Müller cells and by the association of horizontal and amacrine cells. Different experimental studies have reported an activation of Müller cells with consequent hypertrophy in the earlier stages of diabetic retinopathy [28–32]. No histopathologic studies have reported changes in OPL thickness in the early stages of diabetes mellitus. Therefore, the INL/OPL thickening would be mostly due to the changes in INL thickness. INL thickening, never previously reported in vivo, may represent a sign of Müller cells activation which is represented by significant hypertrophy of these cells. Müller cells are particularly susceptible to hyperglycemia and are recognized as key elements in the onset and the progression of retinal damage induced by hyperglycemia [33]. Diabetes induces hypertrophy (swelling) of Müller cells with a limited impact on the apoptotic cascade [34, 35]. Metabolic and morphological alterations of Müller cells induce secondary progressive neuronal loss, due to the crucial role of Müller cells in mediating relationship between retinal vessels and neurons [36–38]. Carrasco et al. have proven that both apoptosis and glial activation precede microvascular lesions, although it is still not known which one of these two events appears first [39, 40]. Müller cells become hyperplastic in DM, with an increasing number of nuclei, as histopathologically demonstrated. In fact, the number of cell nuclei is increased in the INL and reaches a multiplication factor of 1.6 times, at 20 weeks of DM [28]. 

There was not a significant difference in ONL/ELM thickness between diabetics without DR and normal subjects. In diabetics with retinopathy the ONL/ELM was reduced just in the superior macular quadrants. The photoreceptor/RPE layer was not different in thickness between diabetics and normal subjects. Therefore, it seems that outer retina is not significantly influenced by diabetes at least in the early stages of disease, whereas the inner retina is precociously affected. 

In the peripapillary area, although RNFL thickness was reduced in diabetics versus controls, it did not reach statistical and clinical significance, probably due to the fact that in this area small changes are more difficult to be clinically detected because of the high density of retinal nerve fibers [41]. The automatic segmentation of SD-OCT used in this study, although not able to identify any single retinal layer, but rather layers by couple, can be easily used in both the macula and the peripapillary region for the inner and outer retinal thickness analysis in normal subjects and in diabetic eyes. Its use in a more advanced cases of diabetic retinopathy, mostly in macular edema, needs to be further validated. A detection of retina layer thickness changes in diabetic patients without retinopathy or at early stages of retinopathy may also help in the early diagnosis of retinal tissue loss in DM and to better elucidate the pathophysiology of this severe chronic disease. Moreover, as inner and outer retinas appear differently affected, it seems crucial to have the possibility to evaluate the different retinal layers separately.

In conclusion, the thinning of the inner neural retina in diabetic patients without clinically detectable retinopathy and with mild and moderate nonproliferative retinopathy without macular edema is confirmed in vivo using SD-OCT. Retinal thinning is mainly due to the selective thinning of inner retinal layers in the central retina, strongly suggesting an early neuronal loss in DR. The neuronal loss is accompanied (or induced) by Müller cells activation, with increasing thickness in the INL. Automatic intraretinal layering by SD-OCT may be a useful tool to diagnose and monitor early intraretinal changes in diabetic retinopathy.

## Figures and Tables

**Table 1:** Demographic characteristics of the patients.

	Control	Diabetic	Grade of DR	
			No DR	NPDR
Patients, number	50	74	30	44
Mean age, yrs (SD)	55.8 (13.0)	56.4 (12.7)	56.1 (12.8)	55.9 (12.6)
Mean diabetes duration, yrs (SD)	—		5.9 (4.1)	18.6 (10.3)
Mean HbA1c % (SD)	—	8.1 (1.4)	7.8 (0.8)	8.2 (1.5)
Visual acuity, logMAR (SD)	0.003 (0.020)		0.011 (0.039)	0.024 (0.066)
IOP, mmHg (SD)	15.3 (1.9)		16.1 (3.4)	16.8 (3.2)
SE, (SD)	−0.04 (1.5)		0.46 (1.1)	0.42 (1.0)

SD: standard deviation; No DR: diabetic patients without retinopathy; NPDR: non proliferative diabetic retinopathy; IOP: intraocular pressure; SE: spherical equivalent.
